# Evaluation of diagnostic accuracy of urine neutrophil gelatinase-associated lipocalin in patients with symptoms of urinary tract infections: a meta-analysis

**DOI:** 10.3389/fped.2024.1368583

**Published:** 2024-05-22

**Authors:** Yin Zhang, Chen Chen, Mark Mitsnefes, Bin Huang, Prasad Devarajan

**Affiliations:** ^1^ Division of Biostatistics and Epidemiology, Cincinnati Children’s Hospital Medical Center, Cincinnati, OH, United States; ^2^ Division of Nephrology and Hypertension, Cincinnati Children’s Hospital Medical Center, Cincinnati, OH, United States; ^3^ Department of Pediatrics, University of Cincinnati College of Medicine, Cincinnati, OH, United States

**Keywords:** neutrophil gelatinase-associated lipocalin, urinary tract infection, acute kidney injury, acute renal failure, meta-analysis

## Abstract

**Introduction:**

Early and accurate diagnosis of urinary tract infection (UTI) can prevent serious sequelae including chronic kidney disease. Multiple individual studies have identified urine neutrophil gelatinase-associated lipocalin (uNGAL) as a promising biomarker for early diagnosis of UTI. We sought to understand the distribution and diagnostic accuracy of uNGAL values in patients presenting with UTI symptoms.

**Methods:**

Our systematic literature reviews in PubMed, Embase, and Cochrane Reviews up to March 2024, identified 25 studies reporting mean/median, standard deviation/quartiles, and detection limits of uNGAL in symptomatic patients with and without culture-confirmed UTI. Seventeen studies were in children. Meta-analyses were performed using the quantile estimation (QE) method estimating the distributions of uNGAL, which were then compared between the UTI and non-UTI groups for identifying the best cut-off points maximizing the Youden index. Sensitivity analyses were performed on all 25 studies including adult patients.

**Results:**

We found that uNGAL levels were significantly higher in samples with confirmed UTI compared to those without. In pediatric studies, median and 95% confidence interval (CI) of uNGAL values were 22.41 (95% CI of 9.94, 50.54) ng/mL in non-UTI group vs. 118.85 (95% CI of 43.07, 327.97) ng/mL in UTI group. We estimated the cut-off point of 48.43 ng/mL with highest sensitivity (96%) and specificity (97%) in children. Sensitivity analysis including both pediatric and adult studies yielded similar results.

**Discussion:**

The level of uNGAL in symptomatic patients with confirmed UTI is much higher than that reported in patients without UTI. It may be used as a diagnostic tool to identify UTI early among symptomatic patients. The range of uNGAL concentrations and cut-off points reported in subjects with UTI is much lower than that reported in patients with acute intrinsic kidney injury.

**Systematic Review Registration:**

https://www.crd.york.ac.uk/, PROSPERO (CRD42023370451).

## Introduction

A urinary tract infection (UTI) is commonly encountered in humans of all ages. Early and accurate diagnosis can prevent serious sequelae such as sepsis, renal scars, chronic kidney disease, and hypertension (1, 2). However, the current gold standard for the diagnosis is a urine culture, the results of which are typically delayed by at least 48 h. Furthermore, urine culture results may be confounded by sample contamination. The presence of classical UTI symptoms may aid in the initial suspicion; however, UTI symptoms can be nonspecific and misleading, especially in infants and children (1, 2). Currently available point-of-care screening tests lack sufficient accuracy. For example, the leukocyte esterase (LE) test has a reported sensitivity and specificity of 79% and 87% for the diagnosis of culture-positive UTI (3). This implies that by using the LE test, 21% of true UTIs will be missed, leading to delayed treatment, and 13% of subjects will receive a false-positive UTI diagnosis with resultant inappropriate antibiotic prescriptions. Indeed, more than 50% of children who are empirically prescribed antibiotics in the emergency department setting for a suspected UTI were subsequently shown to not have a true UTI (4). The performance of urine WBC count and urinary nitrite is even poorer, with a sensitivity of 74% and 45% respectively (3). Thus, there is an unmet clinical need for a sensitive and specific urinary biomarker that can provide a rapid diagnosis in subjects with UTI symptoms.

In recent years, several urinary biomarkers have been investigated for their diagnostic accuracy for UTI, based on their established role in the host response to inflammatory urinary pathogens (5–7). A contemporary unbiased approach used machine learning algorithms to explore 42 different immunological predictors for a positive urine culture among adult women with UTI symptoms (5). The most promising biomarkers identified were neutrophil gelatinase-associated lipocalin (NGAL), matrix metalloproteinase 9 (MMP9), CXCL8 and interleukin-1β (IL-1β). While other targeted approaches over the years have additionally identified interleukin-8, tumor necrosis factor-α, and urine antimicrobial peptides (5–7) as putative predictors of UTI, the most promising and widely studied UTI biomarker is NGAL (8–12). Pre-clinical studies have established the biological, teleological, and functional roles of NGAL (13–18). There is now a strong rationale for the use of kidney-derived uNGAL as a UTI biomarker, independent of neutrophil presence or activation, in contrast to the LE and pyuria tests (17, 18).

Despite ample biologic plausibility, accumulating human evidence has revealed significant variations in the test characteristics and diagnostic accuracy of uNGAL for the diagnosis of UTI (8–12). In addition, uNGAL has also been widely investigated and advanced as a clinical diagnostic biomarker in other kidney diseases, especially in acute kidney injury (AKI) (19–28). Standard analytical laboratory platforms for the rapid measurement of uNGAL are already available and used globally for the early diagnosis and risk stratification of AKI in some centers. Thus, it is particularly important to understand the distribution of uNGAL values in patients with a possible UTI, to further clarify the use of uNGAL as a clinical diagnostic tool in other kidney conditions. Therefore, in this meta-analysis of all published studies to date, we aimed to determine and compare the distribution of uNGAL in subjects presenting with UTI symptoms. We also sought to determine the overall diagnostic accuracy of uNGAL, by determining the area under the receiver operating characteristic (ROC) curve and the optimal cut-off points for the prediction of UTI.

## Methods

### Study design

The meta-analysis of observational studies in epidemiology (MOOSE, 29) guideline was used to summarize the evidence on value of uNGAL for the diagnosis of UTI. The PRISMA checklist for the reporting of this meta-analysis is shown in Supplementary Table S1.

### Search strategy

This review is registered with PROSPERO (registration number: CRD42023370451). The literature search was performed multiple times and finalized on 03/02/2024, using the electronic bibliographic databases: PubMed, Embase, and Cochrane Reviews. The search keywords were neutrophil gelatinase-associated lipocalin, NGAL, lipocalin, urinary tract infection, UTI, and urine culture. Articles written in English language and published from initiation of the database to March 2024 were retrieved. Studies were included in the analysis if they contained data on the combination of UTI symptoms, urine culture, and uNGAL. Studies reporting on plasma NGAL in UTI were not included in this analysis. Both pediatric and adult populations were included based on the current knowledge that uNGAL levels are similar in all ages beyond the immediate neonatal period. Conference abstracts were excluded. If the same data were reported in multiple studies, only the most comprehensive one was considered. Reference lists were also reviewed and identified for additional articles that may not be searchable in the databases.

### Data extraction

The full text of these studies was screened, and the data were independently summarized by two researchers. A third researcher was involved in discussion to resolve uncertainty about eligibility of study. The primary outcome was the distribution of urine NGAL values in the UTI and non-UTI samples in subjects presenting with UTI symptoms. UTI was identified based on a positive urine culture. Summary statistics of the mean, standard deviation (SD), median, 25%-tile (Q1), 75%-tile (Q3), minimum, maximum, and sample size (n) were extracted from the identified studies separately for the groups with and without UTI. Study types (prospective cross-sectional study, retrospective cross-sectional study, and case-control study), sample characteristics including population, UTI diagnosis, assay type, and publication information (journal, author, year of publication) were also summarized and documented. Missing data and data inquiry were communicated directly with study authors.

### Risk of bias and applicability concerns assessment

The risk of bias and applicability concerns among included studies were assessed based on quality assessment of diagnostic accuracy studies (QUADAS-2) recommendations (30). Two researchers conducted assessments in each study. The characteristics considered were patient selection, index test, reference standard, and flow and timing. Any disagreement was resolved through discussion between researchers.

### Statistical analysis

The uNGAL is not normally distributed and may be subject to detection limits. Studies have therefore often reported median or mean of log transformed NGAL. Studies reported as median and quartiles are often not included in traditional meta-analysis studies utilizing reported means and standard deviations (SD) only. To include studies reporting median and quartiles as well as studies reporting mean and standard deviation, we applied Quantile Estimation (QE) method (31). This approach has gained popularity in meta-analyses of biomarker studies, allowing for the inclusion of a broader range of eligible studies by accommodating different statistical summaries (32–34).

Our primary analysis focused on estimating distributions of uNGAL in pediatric populations with and without symptomatic UTI. We first applied the QE method implemented in the R package estmeansd, to convert all study reports into corresponding mean and standard deviation for both UTI and non-UTI samples. Then, random effect meta-analyses models were applied to estimate averaged mean and SD of log-transformed uNGAL, assuming log-normal distribution. Heterogeneities were reported using Cochran Q statistic and I². Forest plot and funnel plots were generated for the UTI and non-UTI samples. Studies that included more than one non-UTI control samples were treated as two individual control samples. Finally, we estimated the optimal cut off point for uNGAL, and its corresponding sensitivity and specificity, by maximizing the Youden index (35). We conducted several sensitivity analyses by: (1) including both pediatric and adult populations, (2) excluding small UTI studies with sample size *N* < 50, (3) including healthy controls, and (4) excluding high/unclear risk of bias studies. A *p*-value <0.05 was considered statistically significant. All analyses were performed in R version 4.2.2 (package: estmeansd and meta-analysis).

## Results

### Description of studies

**Figure 1:**
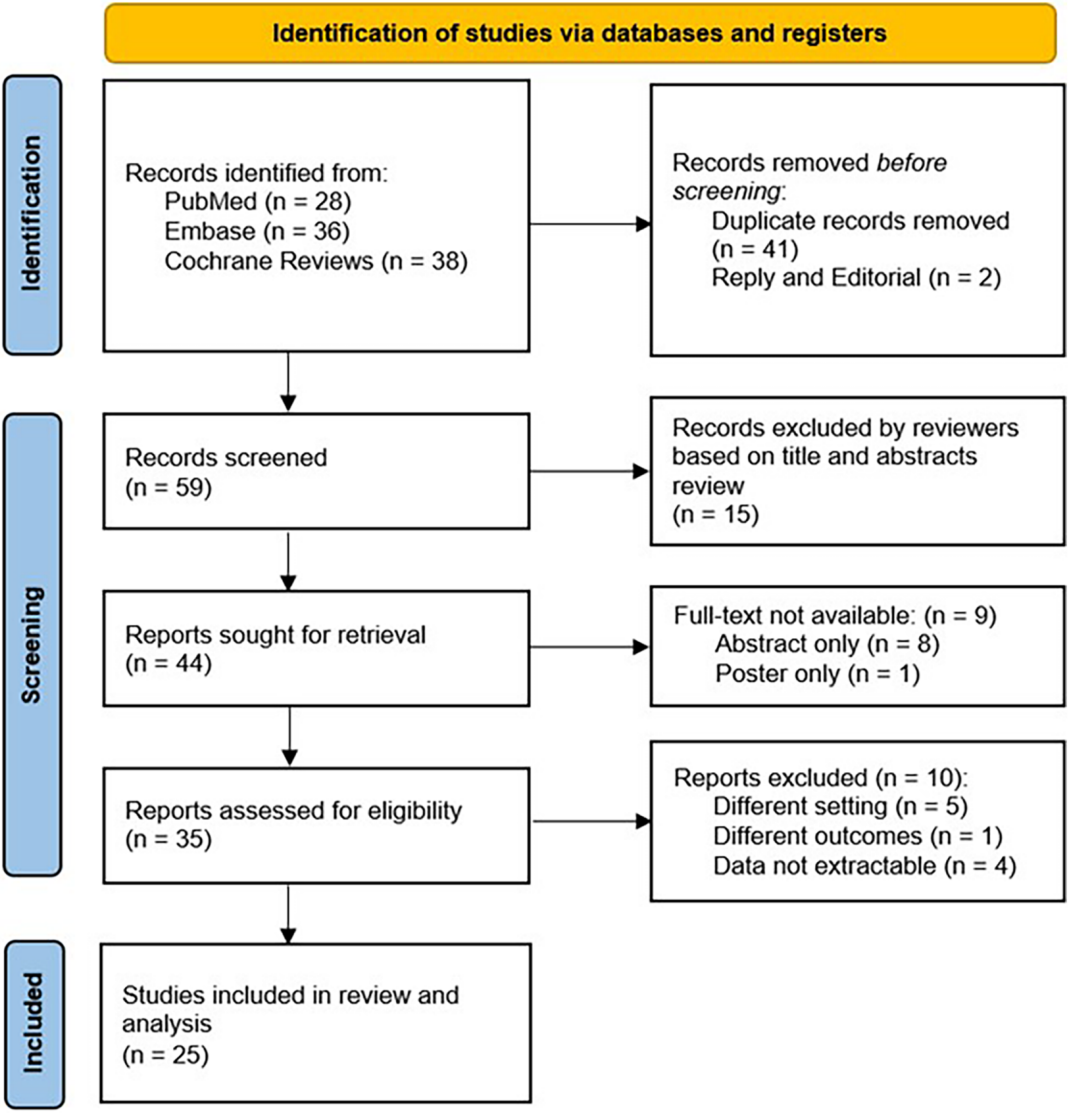
Flow diagram of search strategy.

In total, we identified 102 papers of which 35 full-text manuscripts were reviewed and 25 articles were included in our analyses (Figure 1). There were 17 studies in children and 8 studies in adults. Overall, the study involved 2,985 patients, who had reported acute UTI symptoms with positive urine culture, acute pyelonephritis with positive culture, or recurrent UTIs. None of the patients were reported to have AKI, chronic kidney disease (CKD), or known congenital anatomic anomalies of the kidney or urinary tract. Urine samples were collected by catheter, sterile bag, midstream catch, or suprapubic aspirate. The detailed summary of eligible studies is shown in Table 1.

**Table 1:** Summary of eligible studies.

					Sub group	UTI group		Control group		Healthy control						
Ref	Publication	Population	Sample	Assay		*N*	Statistics	*N*	Statistics	*N*	Statistics	AUC	Cutoff	Sensitivity	Specificity	Method of define Cut off
36	Petrovic 2013	Children	Sterile bag	ELISA (R&D Systems)		50	Mean 155	–		–		–	–	–	–	–
							SD 92.39									
37	Kim 2014	Children	Catheter	ELISA (BioPorto)		284	Mean 19	528	Mean 14.3	–		0.58	5.75	70%	42%	ROC analysis
							SD 44.5		SD 34.6							
38	Urbschat 2014	Adults	Midstream catch	ELISA (R&D Systems)	Upper UTI	30	Mean 111.07	–		38	Mean 49.78	–	–	–	–	–
											SD 58.6					
							SD 114.29									
38	Urbschat 2014	Adults	Midstream catch	ELISA (R&D Systems)	Lower UTI	29	Mean 100.61									
							SD 95.38									
39	Lee 2015	Children	Catheter	ELISA (BioPorto)		33	Median 380	3	Median 16	33	Median 10	Prediction metrics for NGAL/Creatinine ratio only				Youden Index
							IQR 250, 725	3	IQR 6, 39		IQR 1, 28					
40	Nickavar 2016	Children	Various	ELISA (BioPorto)		37	Median 0.48	26	Median 0.065	–		0.75	0.2	76%	77%	Youden Index
							IQR 0.15, 0.72		IQR 0.01, 0.24							
41	Price 2017	Adults	Midstream catch	ELISA (R&D Systems)		50	Median 88.9			50	Median 3.6	0.99	23.9	89%	100%	ROC analysis
							IQR 40.7, 193.4				IQR 2.5, 8.1					
42	Lubell2017	Children	Catheter	ELISA (BioPorto)		35	Median 215.1	225	Median 4.4	–		0.98	39.1	97%	96%	Youden Index
							IQR 100.3, 917.8		IQR 1.6, 11.8							
43	Valdimarsson 2017	Infants	Suprapubic aspirate	ELISA (BioPorto)		108	Median 192	64	Median LOD	13	Median LOD	0.97	38	93%	95%	Youden Index
							IQR 100, 364		IQR LOD, 16.5		IQR LOD, LOD					
44	Forster 2018	Children	Catheter	ELISA (BioPorto)	UTC	24	Median 434	100	Median 135	–		0.88	229	83%	86%	Youden Index
							IQR 309, 969		IQR 54, 224							
44	Forster 2018	Children	Catheter	ELISA (BioPorto)	No growth			77	Median 18							
									IQR 5, 78							
45	Jung 2018	Infants	Bag	Immunoassay (Architect)		102	Median 366.6	320	Median 26.9	–		0.94	46.2	90%	93%	–
							Range 4.5–742.3		Range 0.7–304.4							
46	Krzemien 2018	Infants	Catheter	ELISA (BioVendor)		42	Mean 67.5	24	Mean 48	18	Mean 38	0.76	42.2	74%	72%	–
							SD 34.8		SD 41.1		SD 32.3					
5	Gadalla 2019	Adults	Midstream catch	ELISA (Mologic)		79	Median 45.25	104	Median 22.63	–		–	–	–	–	–
							Range 1–59.7		Range 1–59.7							
47	Forster 2020	Children	Catheter	ELISA (BioPorto)	Single UTI	44	Median 456	6	Median 37	–		0.96	For Creatinine standardized NGAL only			–
							IQR 214, 779		IQR 19, 52							
47	Forster 2020	Children	Catheter	ELISA (BioPorto)	Recurrent UTI	20	Median 395									
							IQR 144, 463									
48	Shaikh 2020	Children	Catheter	ELISA (Thermofisher)		111	Mean 620.49	89	Mean 140.02	–		0.89	–	79%	90%	Youden Index
							SD 554.54		SD 337.84							
49	Forster 2021	Adults with NLUTD	Catheter	ELISA (BioPorto)	Unlikely UTI	8	Median 187	67	Median 95	–		–	–	–	–	–
							IQR 146, 224		IQR 37, 161							
49	Forster 2021	Adults with NLUTD	Catheter	ELISA (BioPorto)	No UTI			29	Median 37							
									IQR 15, 71							
50	Lubell 2022	Children	Catheter	ELISA (BioPorto)	Possible Positive UTI	21	Median 210.1	7	Median 16.5	–		0.96	39.1	91%	94%	Youden Index
							IQR 95.8, 1000		IQR 12, 162.1							
50	Lubell 2022	Children	Catheter	ELISA (BioPorto)	Culture Negative			183	Median 0.01							
									IQR 0.01, 11.6							
51	Moon 2021	Children	Catheter	Immunoassay (Architect)		157	Mean 240.9	164	Mean 31.6	–		0.86	36.5	82%	79%	–
							SD 292.4		SD 63.1							
52	Pamuk 2022	Children	Catheter	ELISA (BioVendor)	Lower UTI	30	Median 30.3	–		28	Median 4.16	0.95	15.6	92%	86%	–
							IQR 22.57, 61.73				IQR 1.71, 11.74					
52	Pamuk 2022	Children	Catheter	ELISA (BioVendor)	Pyelonephritis	29	Median 66.15									
							IQR 25.66, 87.61									
53	Shaikh 2022	Children	Catheter	ELISA (Thermofisher)		75	Mean 419.99	69	Mean 8	48	Mean 4.3775	1	39.1	100%	97%	Youden Index
							SD 134.2		SD 40.7		SD 21.4					
54	Haley 2023	Adults	–	ELISA (R&D Systems)	M-PCR+/SUC- 10K	86	Median 117.4	120	Median 14.47			The optimal cut-off point was determined using thresholds presented previously in the literature (38.0)				
							IQR 13.9, 299.2		IQR 0.2, 40.7							
54	Haley 2023	Adults	–	ELISA (R&D Systems)	M-PCR-/SUC + 10K	26	Median 12.79									
							IQR 1.02, 89.51									
54	Haley 2023	Adults	–	ELISA (R&D Systems)	Both positive 10K	351	Median 201.75									
							IQR 57.46, 489.6									
55	Kim 2023	Children	Catheter	Immunoassay (Architect)		218	Median 204.5	238	Median 33			0.74	68.4	Subgroups only		AUC after dichotomize
									IQR 8.7, 104							
							IQR 87.9, 461.6									
56	Parnell 2023	Adults	Midstream catch	ELISA (R&D Systems)	No Microbes Detected			117	Median 16.05			Previously published thresholds for biomarker positivity were used as cut-off point (38.0)				
									IQR 0.2, 50.4							
56	Parnell 2023	Adults	Midstream catch	ELISA (R&D Systems)	<10,000 cells/ml			23	Median 34.55							
									IQR 8.75, 114.25							
56	Parnell 2023	Adults	Midstream catch	ELISA (R&D Systems)	10,000–99,999 cells/ml	79	Median 53.8									
							IQR 12.8, 231									
56	Parnell 2023	Adults	Midstream catch	ELISA (R&D Systems)	≥100,000 cells/ml	364	Median 228.9									
							IQR 75.2, 494.6									
57	Shaikh 2023	Children	Catheter	ELISA (BioPorto)		50	Mean 326.5	324	Mean 16.7			0.96	39.93	90%	96%	Youden Index
							SD 258.6		SD 67.9							
58	Akhlaghpour 2024	Adults	Midstream catch	ELISA (R&D Systems)	Symptomatic, SUC and M-PCR +	351	Mean 251.8					Previously published thresholds for biomarker positivity were used as cut-off point (38.0)				
							SD 193.9									
							Median 211									
							IQR 64.6, 500									
58	Akhlaghpour 2024	Adults	Midstream catch	ELISA (R&D Systems)	Asymptomatic, No Microbes					110	Mean 4.2					
											SD 11.3					
											Median 0.16					
											IQR 0.16, 0.16					
58	Akhlaghpour 2024	Adults	Midstream catch	ELISA (R&D Systems)	Asymptomatic, SUC or M-PCR +					118	Mean 24.4					
											SD 56.8					
											Median 0.16					
											IQR 0.16, 17.7					
58	Akhlaghpour 2024	Adults	Midstream catch	ELISA (R&D Systems)	Asymptomatic, SUC and M-PCR +					51	Mean 36.5					
											SD 70.2					
											Median 9.5					
											IQR 0.16, 27					
59	Bilsen 2024	Adults	Midstream catch	LC-MS		62	Median 594			100	Median 59	0.86	201	87%	72%	Youden Index
							IQR 289, 1772									
											IQR 20, 234					

### Risk of bias and applicability concerns

**Figure 2:**
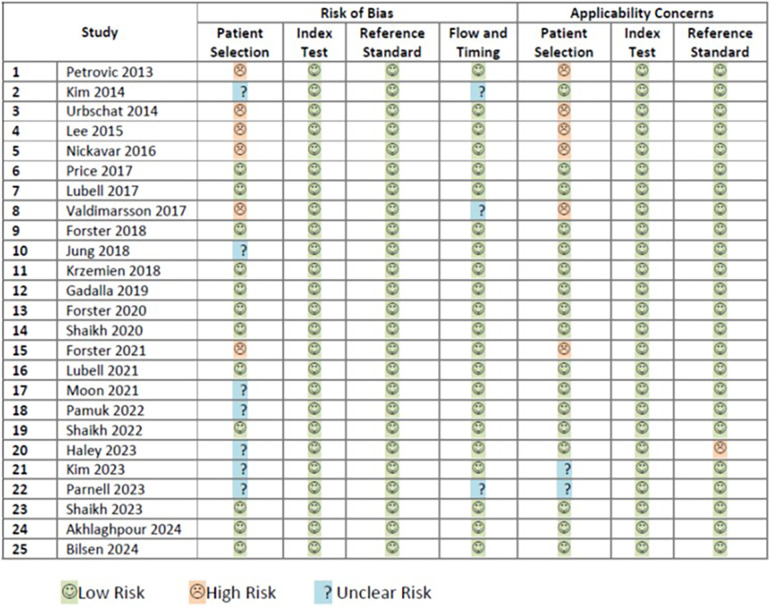
Rating of risk of biases and applicability concerns.

The risk of bias assessment of 25 included studies (5, 36–59) was performed according to the guidance of QUADAS-2. Twelve studies were rated as having low risk of all four domains of bias and were classified as low risk of bias (5, 41, 42, 44, 46–48, 50, 53, 57–59). Six studies were classified as having high risk of bias (36, 38–40, 43, 49). Seven remaining studies had unclear risk of bias (37, 45, 51, 52, 54–56). Details on the rating of each risk of bias are shown in Figure 2.

The risk of applicability concerns assessment of the same 25 included studies (5, 36–59) was also performed according to the guidance of QUADAS-2. Sixteen studies were rated as having low risk of all three domain of applicability concerns (5, 37, 41, 42, 44–48, 50–53, 57–59). Seven studies were classified as having high risk of applicability concerns (36, 38–40, 43, 49, 54). Two studies had unclear risk of applicability concern (55, 56). Details on the rating of each applicability concern domain are shown in Figure 2.

### Quantitative analysis: non-UTI samples in pediatric studies

**Figure 3:**
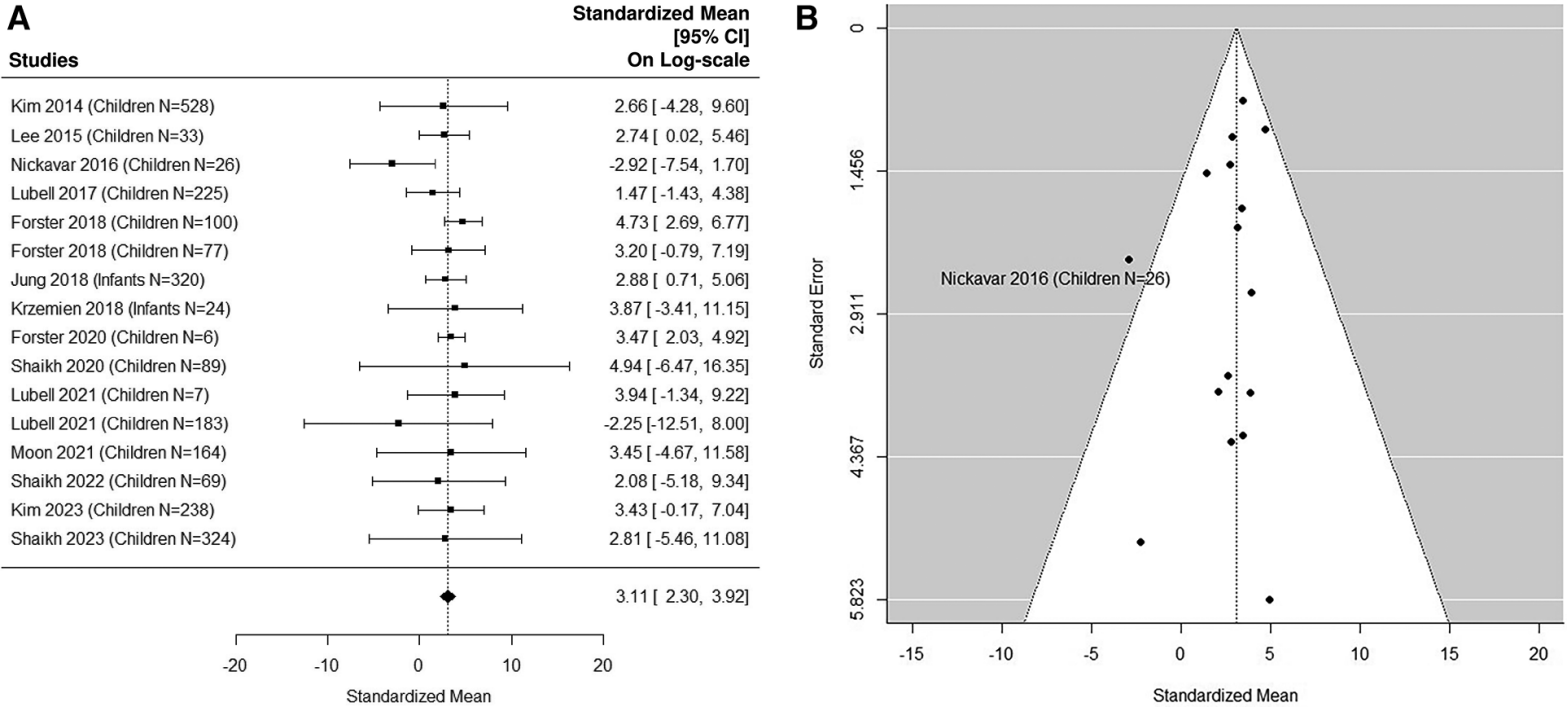
(**A**) forest plot of log_e_-transformed NGAL values for non-UTI samples in pediatric studies. Each row corresponds to a non-UTI group, displaying the standardized mean on a logarithmic scale along with its 95% confidence intervals (CIs). The diamond symbol represents the mean average of log_e_-transformed NGAL values. Upon applying anti-log transformation to revert NGAL values to their original scale in ng/ml, the calculated mean average (95% CI) is 22.42 (9.97, 50.40) ng/ml. (**B**) Funnel plot of publication bias for non-UTI samples in pediatric studies. The horizontal axis is standardized log-NGAL and the vertical axis is standard error of log-NGAL. The diagonal lines represent the 95% confidence limits of estimation.

The forest plot shown in Figure 3A summarizes the log transformed uNGAL value in each study for the non-UTI samples reported in the pediatric studies. 16 non-UTI groups from 14 studies were included (37, 39, 40, 42, 44–48, 50, 51, 53, 55, 57). Summarized mean and standard deviation were reported. The results suggested that the median uNGAL value in the non-UTI children population is 22.41 (95% CI of 9.94, 50.54) ng/ml. No potential heterogeneous issue was detected, with Cochran Q statistic 11.98 (15 DF), *P* = 0.68. The *I*^2^ of 0% was also statistically insignificant. Details are shown in funnel plots (Figure 3B).

### Quantitative analysis: UTI samples in pediatric studies

**Figure 4:**
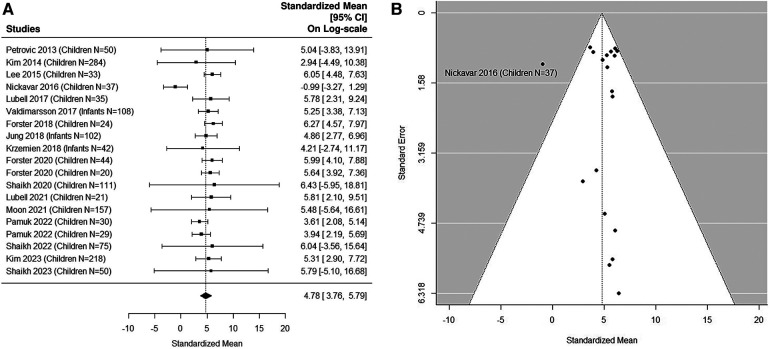
(**A**) forest plot of log_e_-transformed NGAL values for UTI samples in pediatric studies. Each row corresponds to a UTI group, displaying the standardized mean on a logarithmic scale along with its 95% confidence intervals (CIs). The diamond symbol represents the mean average of log_e_-transformed NGAL values. Upon applying anti-log transformation to revert NGAL values to their original scale in ng/ml, the calculated mean average (95% CI) is 119.10 (42.95, 327.01) ng/ml. (**B**) Funnel plot of publication bias for UTI samples in pediatric studies. The horizontal axis is standardized log-NGAL and the vertical axis is standard error of NGAL. The diagonal lines represent the 95% confidence limits of estimation.

The forest plot shown in Figure 4A summarizes the log transformed uNGAL value in each study for the UTI samples reported in the pediatric studies. 17 pediatric studies contributed 19 UTI groups of patients (36, 37, 39, 40, 42–48, 50–53, 55, 57). Summarized mean and standard deviation were reported. The results suggested that the median uNGAL value is 118.85 (95% CI of 43.07, 327.97) ng/ml in the UTI patient population. We did find statistically significant Cochran Q statistic 37.17 (18 DF), *P* = 0.005 and large *I*^2^ (59.3%) indicating heterogeneous results reported from different studies. Details are shown in funnel plots (Figure 4B).

### Optimal cut-off point

The estimated area under the curve (AUC) is 0.99. We estimated that the cut-off point of 48.43 ng/ml would optimize Youden Index with sensitivity of 96% and specificity of 97% (36, 37, 39, 40, 42–48, 50–53, 55, 57).

### Sensitivity analysis 1: inclusion of both pediatric and adult studies

**Figure 5:**
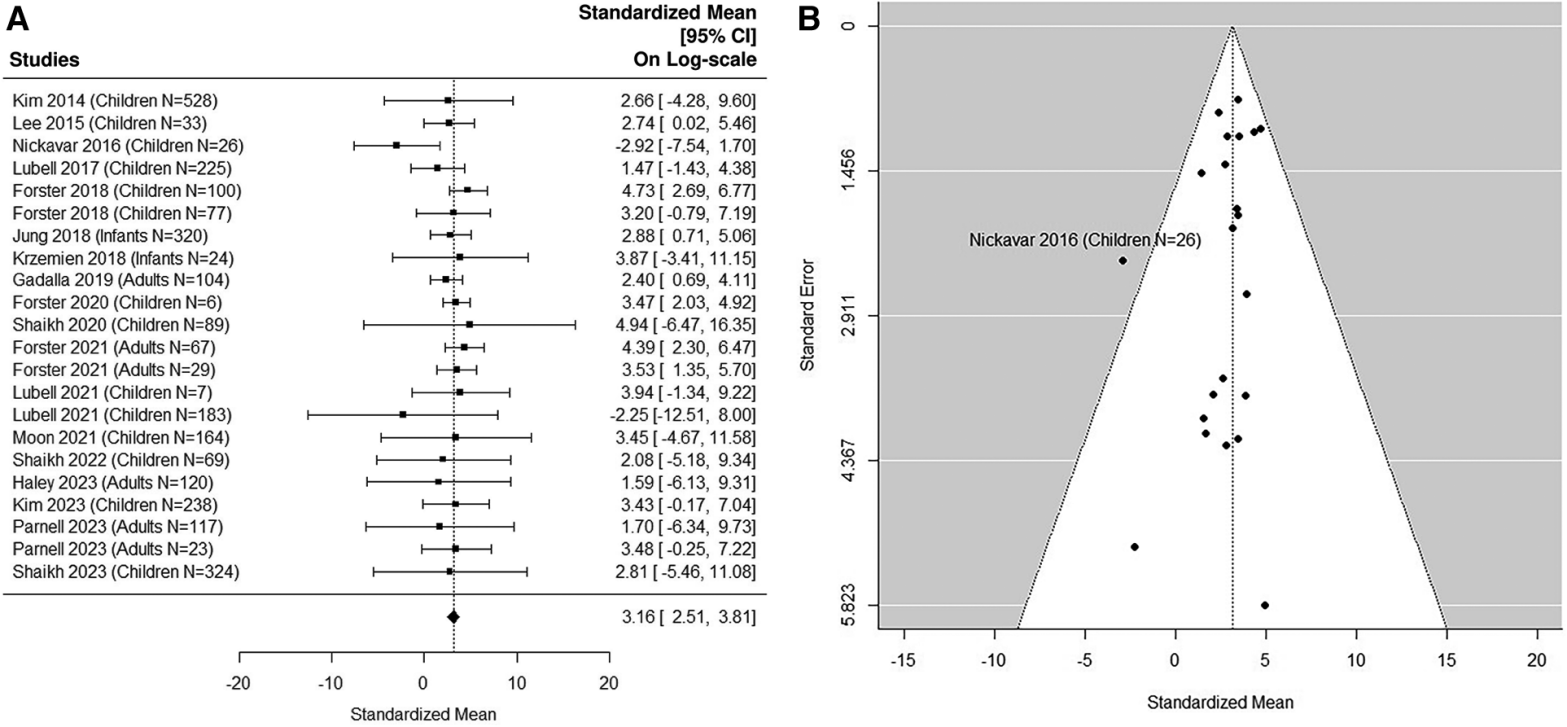
(**A**) forest plot of log_e_-transformed NGAL values for non-UTI samples in sensitivity analysis including both pediatric and adult studies. Each row corresponds to a non-UTI group, displaying the standardized mean on a logarithmic scale along with its 95% confidence intervals (CIs). The diamond symbol represents the mean average of log_e_-transformed NGAL values. Upon applying anti-log transformation to revert NGAL values to their original scale in ng/ml, the calculated mean average (95% CI) is 23.57 (12.30, 45.15) ng/ml. (**B**) Funnel plot of publication bias for non-UTI samples in sensitivity analysis including both pediatric and adult studies. The horizontal axis is standardized log-NGAL and the vertical axis is standard error of NGAL. The diagonal lines represent the 95% confidence limits of estimation.

**Figure 6:**
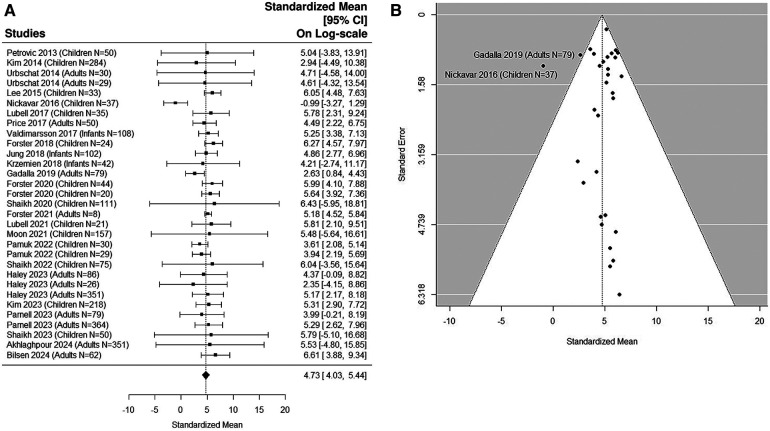
(**A**) forest plot of loge-transformed NGAL values for UTI samples in sensitivity analysis including both pediatric and adult studies. Each row corresponds to a UTI group, displaying the standardized mean on a logarithmic scale along with its 95% confidence intervals (CIs). The diamond symbol represents the mean average of log_e_-transformed NGAL values. Upon applying anti-log transformation to revert NGAL values to their original scale in ng/ml, the calculated mean average (95% CI) is 113.30 (56.26, 230.44) ng/ml. (**B**) Funnel plot of publication bias for UTI samples in sensitivity analysis including both pediatric and adult studies. The horizontal axis is standardized log-NGAL and the vertical axis is standard error of NGAL. The diagonal lines represent the 95% confidence limits of estimation.

8 adult studies (5, 38, 41, 49, 54, 56, 58, 59) were added in the sensitivity analysis (Figures 5A,B, 6A,B). The median uNGAL value in the non-UTI patient population is 23.56 (95% CI of 12.35, 44.93) ng/ml. The median uNGAL value in the UTI patient population is 113.73 (95% CI of 56.13, 230.45) ng/ml. We estimated that the cut-off point of 50.31 ng/ml would optimize Youden Index with sensitivity of 99% and specificity of 99% when both pediatric and adult studies were included.

### Sensitivity analysis 2: excluding small UTI studies

**Figure 7:**
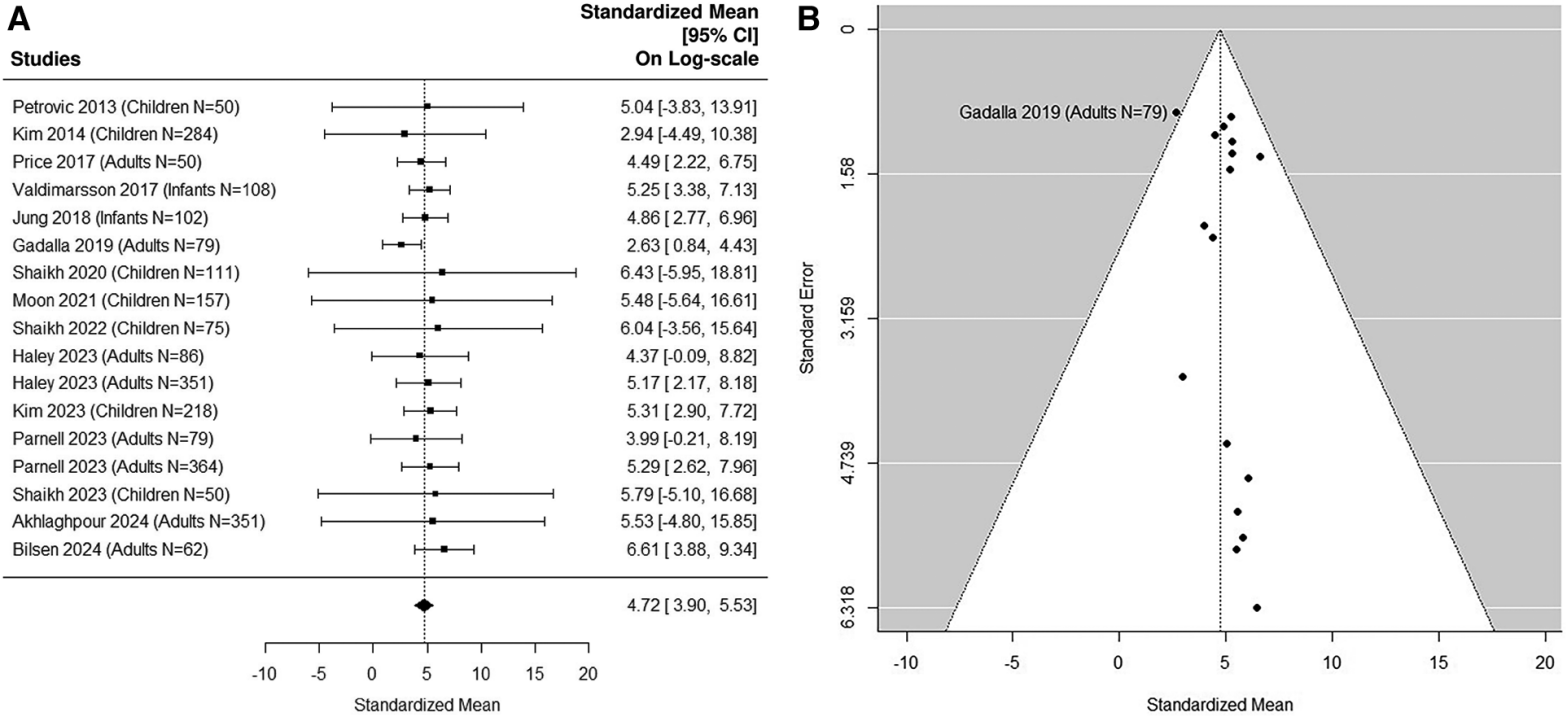
(**A**) forest plot of log_e_-transformed NGAL values for UTI samples in sensitivity analysis excluding small UTI groups. Each row corresponds to a UTI group, displaying the standardized mean on a logarithmic scale along with its 95% confidence intervals (CIs). The diamond symbol represents the mean average of log_e_-transformed NGAL values. Upon applying anti-log transformation to revert NGAL values to their original scale in ng/ml, the calculated mean average (95% CI) is 112.17 (49.40, 252.14) ng/ml. (**B**) Funnel plot of publication bias for UTI samples in sensitivity analysis excluding small UTI groups. The horizontal axis is standardized log-NGAL and the vertical axis is standard error of NGAL. The diagonal lines represent the 95% confidence limits of estimation.

Fifteen studies (5, 36, 37, 41, 43, 45, 48, 51, 53–59) were included in the sensitivity analysis after excluding small UTI studies with sample size *N* < 50 (Figures 7A,B). The Cochran Q statistic 8.5 (16 DF), *P* = 0.93 and *I*^2^ = 7.76% indicating small heterogeneity. The median estimated uNGAL is 111.77 (95% CI of 49.4, 252.7) ng/ml.

### Sensitivity analysis 3: including healthy control groups

Eight studies (38, 39, 41, 46, 52, 53, 58, 59) with healthy control groups were added in the non-UTI analysis. The median uNGAL value in the non-UTI patient population is 19.56 (95% CI of 10.94, 34.94) ng/ml. The Cochran Q statistic 18.6 (31 DF) and *I*^2^ of 0% are statistically insignificant. No potential heterogeneous issue was detected.

### Sensitivity analysis 4: excluding studies with high/unclear risk of bias

Twelve studies (5, 41, 42, 44, 46–48, 50, 53, 57–59) were rated as having low risk of all four domains of bias and were included in analysis. In the non-UTI patient population, The Cochran Q statistic 5.9 (10 DF), *P* = 0.83, and *I*^2^ of 0.68% are statistically insignificant. No potential heterogeneous issue was detected. The median uNGAL value in the non-UTI group is 24.42 (95% CI of 10.23, 58.30) ng/ml. In the UTI patient population, the Cochran Q statistic 12.1 (12 DF), *P* = 0.44, and *I*^2^ of 30.2% indicated moderate heterogeneous results reported from different studies. The median uNGAL value in the UTI patient population is 197.98 (95% CI of 72.97, 537.17) ng/ml. We estimated that the cut-off point of 65.69 ng/ml would optimize Youden Index with sensitivity of 98% and specificity of 98%.

## Discussion

The use of uNGAL as a diagnostic tool for UTI has been the subject of several studies in recent years. In this review, we aimed to estimate the distribution of uNGAL levels in patients with reported UTI symptoms, comparing samples with culture-confirmed UTI vs. those without UTI. Our meta-analysis found that uNGAL levels were significantly higher in samples with confirmed UTI compared to those without. Median uNGAL values are 22.41 (95% CI of 9.94, 50.54) ng/ml in the non-UTI group vs. 118.85 (95% CI of 43.07, 327.97) ng/ml in UTI group in the primary analysis of pediatric studies. We estimated the optimal cut-off point of 48.43 ng/ml with high sensitivity (96%) and specificity (97%). Sensitivity analyses by including both pediatric and adult studies, by including healthy control in the non-UTI group, and by excluding studies with small sample sizes provided results consistent with the primary analysis. When both the 17 pediatric studies and 8 adult studies were analyzed, results were comparable to the primary pediatric analysis. The median uNGAL value in the non-UTI pediatric patient population is 23.56 (95% CI of 12.35, 44.93) ng/ml. The median uNGAL value in the UTI pediatric patient population is 113.73 (95% CI of 56.13, 230.45) ng/ml. In all patients, we estimated that the cut-off point of 50.31 ng/ml would optimize Youden Index with sensitive (99%) and specificity (99%).

Overall, our results support the use of uNGAL as a potential biomarker for diagnosing UTI in both pediatric and adult populations. In a previous smaller meta-analysis of 12 published studies in children and adolescents, Abbasi et al. (9) compared means and standard deviations between UTI and non-UTI groups and summarized the findings using standardized mean difference. They suggested that 30–39.9 ng/ml can be used as optimal cut-off point, with sensitivity and specificity of 0.89 (95% CI of 0.64, 0.97) and 0.89 (95% CI of 0.71, 0.97) respectively, which is similar to our findings in this larger meta-analysis. Shaikh et al. (11) focused on accuracy values from 12 published studies and examined how accuracy varied with threshold. Recognizing that the distribution of uNGAL is highly skewed and subject to the limit of detections in different studies, our study applied the QE method to mitigate the issues. We first evaluated the distributions of uNGAL in the UTI and non-UTI samples, then estimated the optimal cut-off points for uNGAL based on the estimated distributions.

Our results are consistent with the current literature, including three studies that presented systematic reviews of smaller groups of publications, but without conducting quantitative meta-analysis. Shaikh et al. (11) compared the performance of uNGAL with the currently used leukocyte esterase (LE) test in diagnosing UTI in febrile children aged 0–18 years. Their study included a review of 4 previously published studies and suggested that uNGAL offered a promising and more sensitive alternative to the LE test for diagnosing UTI. Martino et al. (12) conducted a systematic review of 4 published studies in an adult population and provided a summary of the results indicating encouraging performance of uNGAL. Horvath et al. (10) summarized 16 studies on uNGAL for predicting UTI in both pediatric and adult population. They suggested that use of uNGAL could improve the sensitivity and specificity of laboratory diagnosis of UTIs.

Pre-clinical studies have established the biological plausibility for the use of uNGAL as a UTI biomarker. In response to inflammation or injury, NGAL is expressed and released from activated neutrophils, as well as from several organs and tissues, especially kidney tubule cells. Within the urinary tract, NGAL plays an essential role in innate immunity and bacteriostasis via its profound iron-chelating properties (13). NGAL-deficient mice are highly susceptible to bacterial infections and die of sepsis when infected with uropathogenic *E. coli* (14). Furthermore, uNGAL is dramatically increased with gram-negative UTIs in several animal models (14, 15). Earlier studies suggested that uNGAL in UTIs may be derived primarily from activated neutrophils in the kidney and urothelium, since a correlation between urinary WBC count and measured uNGAL concentrations was demonstrated (16). However, subsequent investigations have illustrated that the alpha-intercalated cells in the kidney collecting duct are the primary source of uNGAL in response to infection or injury (17, 18). Depletion of neutrophils did not affect kidney NGAL expression in mice, and isolated cultured primary kidney tubule cells robustly upregulate NGAL in response to uropathogenic *E. coli* in the complete absence of neutrophils (17). In addition, specific ablation exclusively of alpha-intercalated cells suppressed uNGAL levels, and impaired bacterial clearance following transurethral inoculation of uropathogenic *E. coli* (18). Collectively, the pre-clinical data strongly support the utility of kidney-derived uNGAL as a biomarker of UTI, independent of neutrophil presence or activation.

The published literature has identified important advantages to the use of uNGAL as a UTI biomarker over other diagnostic methods. For example, Kim et al. studied 218 children with culture-positive UTI and showed that urine specific gravity did not affect the diagnostic performance of uNGAL, and that uNGAL consistently demonstrated higher AUCs compared to pyuria in both dilute and concentrated urine samples (55). In a prospective study of febrile children being evaluated for UTIs with paired catheterized and bagged urine samples, bagged sample uNGAL had lower quantitative specificities (73.8%) than from catheterized samples (94.3%), although the AUC for the positive diagnosis of UTI was comparable in paired catheterized and bagged urine samples, at 0.96 (95% CI = 0.89–1.00) and 0.93 (95% CI = 0.87–0.99) respectively (50). Therefore, while bagged specimens can be confounded by contamination issues in young children, the lower specificity for uNGAL in bagged vs. catheterized specimens reported in this study should be taken into consideration and is worthy of further characterization. In older children and adults, uNGAL can be reliably measured in voided urine samples, although one study found that both uNGAL levels and WBC counts were higher in initial-stream urine samples in comparison with midstream, leading to a recommendation that midstream urine sampling is desirable for uNGAL measurements independent of pyuria (60). In children with neurogenic bladders who are at high risk for UTIs, an elevated uNGAL reliably differentiated culture positive UTIs from either urinary tract colonization (44) or from asymptomatic bacteriuria (61), allowing in both instances to safely withhold unnecessary antibiotic treatment.

It is important to note that our review and meta-analysis had some limitations. Firstly, there was significant heterogeneity in the included studies in terms of study design, study population, sample size, and assay type. This heterogeneity may have influenced the accuracy of our meta-analysis results. Secondly, while we included studies from both pediatric and adult populations, most studies included were conducted in children. More research is needed to better establish the diagnostic value of uNGAL in age and/or gender specific populations. Third, the UTI and non-UTI samples were not necessarily matched by demographics. Future studies may wish to use the individual patient pooled data for better analyses adjusting for potential confounding bias. Fourth, we did not include studies that examined plasma NGAL for the diagnosis of UTI, due to the relatively few numbers of publications on that subject (36, 46, 62–64). Fifth, Western blotting studies have revealed two forms of uNGAL in patients with UTI, including a monomeric (25 kDa) forms produced primarily by the kidney tubule epithelial cells and a homodimeric (45 kDa) form that emanates from activated neutrophils (65). Although the studies reported herein utilized sensitive ELISA or standard clinical immunoassays for uNGAL quantification, it is unclear if the assays preferentially detect one or both forms, which might affect their accuracy. Therefore, it is likely that the uNGAL detected in patients with UTI using ELISA or standardized laboratory platforms might represent both the kidney-specific monomeric 25 kDa form as well as other homodimeric forms from activated neutrophils.

Finally, it should be acknowledged that uNGAL has also been used as a clinical diagnostic biomarker in other kidney states, especially in AKI (19–28). Indeed, in 2023, the FDA approved uNGAL as a clinical laboratory test (ProNephro AKI^™^) for use in critically ill patients aged 3 months through 21 years as an aid in the risk assessment for moderate or severe AKI within 48–72 h of ICU admission (66). However, the range of uNGAL concentrations and cut-off points reported in subjects with AKI has generally been much higher than the 48.43 ng/ml reported herein for UTIs. For example, an initial meta-analysis of 19 studies that measured uNGAL using ELISA techniques identified a cut-off point range of 100–270 ng/ml for optimal sensitivity and specificity to predict AKI (22). A subsequent evaluation of 58 published studies suggested an NGAL cut-off point of >150 ng/ml, measured on a standardized clinical laboratory platform, as diagnostic for AKI (27). This summary of studies reporting on uNGAL cut-off points concentrations for AKI found a range from ≥105 to ≥350 ng/ml (27). In a recent meta-analysis of individual study data from 30 publications reporting on uNGAL measurements for AKI prediction using clinical laboratory platforms, a cut-off points concentration of 105 ng/ml yielded the optimal combination of sensitivity and specificity, and a cut-off point of >580 ng/ml provided 95% specificity (28). Nevertheless, clinicians should be aware of some overlap between uNGAL values between the two conditions (UTI and AKI) and exert appropriate caution when interpreting patient-specific results.

In conclusion, our results confirm that uNGAL can accurately distinguish patients with and without confirmed UTI among symptomatic patients with no AKI, CKD, or known congenital anatomic anomalies of the kidney or urinary tract. Future prospective studies to confirm the identified uNGAL cut-off point of 48.43 ng/ml for early UTI diagnosis are warranted.

## Data Availability

The original contributions presented in the study are included in the article/**Supplementary Material**, further inquiries can be directed to the corresponding author.
